# Effectiveness of Oil Pulling for Improving Oral Health: A Meta-Analysis

**DOI:** 10.3390/healthcare10101991

**Published:** 2022-10-11

**Authors:** Tzu-Rong Peng, Han-Yu Cheng, Ta-Wei Wu, Boon-Kok Ng

**Affiliations:** 1 Department of Pharmacy, Taipei Tzu Chi Hospital, Buddhist Tzu Chi Medical Foundation, New Taipei City 231405, Taiwan; 2 Department of Dentistry, Taipei Tzu Chi Hospital, New Taipei City 231405, Taiwan

**Keywords:** oil pulling, effectiveness, oral hygiene, dental health

## Abstract

**Objective:** The effect of oil pulling on oral health has not yet been fully demonstrated. Therefore, we performed a meta-analysis to investigate the effect of oil pulling on oral health. **Methods:** We searched PubMed, the Cochrane Library, and the EMBASE database, limiting the search to human patients and articles written in English and published before 31 July 2022. We included randomized controlled trials (RCTs) comparing the effect of oil pulling on improving dental health and oral hygiene. The outcomes of this study were salivary bacteria count, plaque index, and gingival index. **Results:** In total, nine RCTs were included in this study. The study showed that salivary bacterial colony (BC) counts were significantly reduced in the oil pulling group compared to the control group [mean difference (MD): 17.55, 95% CI 2.56, 32.55]. There was no significant difference between the two groups (MD: −0.10, 95% CI −0.33, 0.14; −0.05, 95% CI −0.12, 0.02) in plaque index and gingival index score. **Conclusions:** Based on the results of this meta-analysis, the oil pulling may have a beneficial effect on reducing salivary BC count compared to the control group. There was no significant difference in the plaque index and gingival index score between the oil pulling and the control group. Therefore, future clinical trials should be more rigorous and better reported.

## 1. Background

Oil pulling is a traditional Ayurvedic remedy originally used in ancient India to maintain oral health. Ayurveda uses a natural approach to herbs and spices, using holistic remedies to find the root cause of any problem and manage it holistically. With people preferring natural remedies over chemical-based allopathic medicines, Ayurveda has truly become the medical method of choice for general wellbeing and health [1]. Ayurveda has several proven practices; oil pulling is one of them. Oil pulling may be a method of oral health. Oil pulling is also believed to improve gingival health and bleeding by reducing inflammation, relieving dry mouth, throat, and chapped lips, whitening teeth, reducing bad breath, and improving oral hygiene [2]. Oil pulling is a method of gargling through oil, allowing the oil to shuttle between the teeth [2]. Oil pulling is best done in the morning on an empty stomach. The recommended dose for adults is one tablespoon (about 10 mL) of sesame oil, sip it between the teeth for about 15-20 min, and then spit it out. The oil sip is sucked and pulled in the mouth for the recommended time, then the viscous oil becomes milky white and thin.

The organic oils used include sunflower oil, sesame oil, and coconut oil [2]. Coconut oil has 92% saturated medium-chain fatty acids, most of which are lauric acid, followed by other acids, such as capric acid, caprylic acid, etc., and its glycolipid component is sucrose monolaurate. It can oxidize sucrose on *Streptococcus mutans*, preventing its regeneration and reattachment of plaque, and has anti-caries properties [3,4]. Previous in vitro studies using a biofilm model have demonstrated the efficacy of coconut oil against *Streptococcus mutans* and *Candida albicans* [5].

In some literature, the effect of oil pulling has not been fully demonstrated [6,7,8], although some studies have provided systematic reviews or quantitative meta-analyses of oil pulling methods for coconut oils [9,10]. However, there is still no clear understanding of the quantitative analysis of oral health effects of the organic oils commonly used in oil pulling. Oil pulling is a simple and available treatment; therefore, the purpose of this review is a systematic quantitative analysis and evaluation of published randomized trials to investigate the effect of oil pulling on oral health.

## 2. Materials and Methods

### 2.1. Search Strategy and Study Selection

We searched PubMed, the Cochrane Library, and the EMBASE database, limiting the search to human patients and articles in English published before 31 July 2022. The following search terms were included in the search: (oil pulling OR oil swishing OR oil gargling OR sesame seed oil OR sunflower oil OR coconut oil OR olive oil OR corn oil) and (dental). All retrieved abstracts, studies, and citations were reviewed. Additionally, we searched the reference sections of the selected papers for relevant studies. Detailed information on the search strategy for eligible studies is given in the flowchart provided by Preferred Reporting Items for Systematic Reviews and Meta-Analyses (PRISMA) [11]. The retrieved studies were independently reviewed by two reviewers (T. R. P. and H. Y. C.). Any discrepancies between the reviewers were resolved by reaching a consensus.

### 2.2. Data Collection and Inclusion Criteria

This study was performed by Cochrane Collaboration guidelines [12]. The following information was extracted: author, year of publication, study design, number of enrolled patients, intervention, and clinical efficacy. Trials that met the following criteria were included: (1) randomized control trial; (2) Patients with plaque, gingivitis, and caries; (3) Intervention with oil pulling as a preventive and therapeutic agent in the management of high plaque, gingiva, and caries indices scores; and (4) Participants using placebo or any other agent used for comparison with coconut oil. No restriction in the publication year of the studies was implemented.

### 2.3. Methodological Quality Appraisal

Two reviewers (T. R. P. and H. Y. C.) independently assessed the methodological quality of each study by using the revised risk-of-bias (version 2.0) method, according to the recommendation of the Cochrane Collaboration [13]. Several domains were assessed, including the adequacy of randomization, allocation concealment, blinding of patients and outcome assessors, length of follow-up, the information provided to patients regarding study withdrawal, whether intention-to-treat analysis was performed, and freedom from other biases.

### 2.4. Statistical Analyses

Statistical analysis was performed according to the Cochrane Handbook for Statistical Review of Interventions (version 5.3) [14]. The meta-analysis was performed using RevMan software (The Cochrane Collaboration, Oxford, UK). Mean difference (MD) with a 95% confidence interval (CI) on continuous outcomes was estimated by employing a random-effects model. The Cochran *Q* test and *I*^2^ statistics were used to assess statistical heterogeneity and inconsistency. Statistical significance was set at *p* < 0.10 for Cochrane *Q* tests. Heterogeneity was considered low, moderate, or high, if the *I*^2^ values was <25%, 25–50%, and >50%, respectively. Results were considered statistically significant when the p-value was less than 0.05. Publication bias was examined by using funnel plots.

## 3. Results

### 3.1. Characteristics of Included Trials

**Figure 1:**
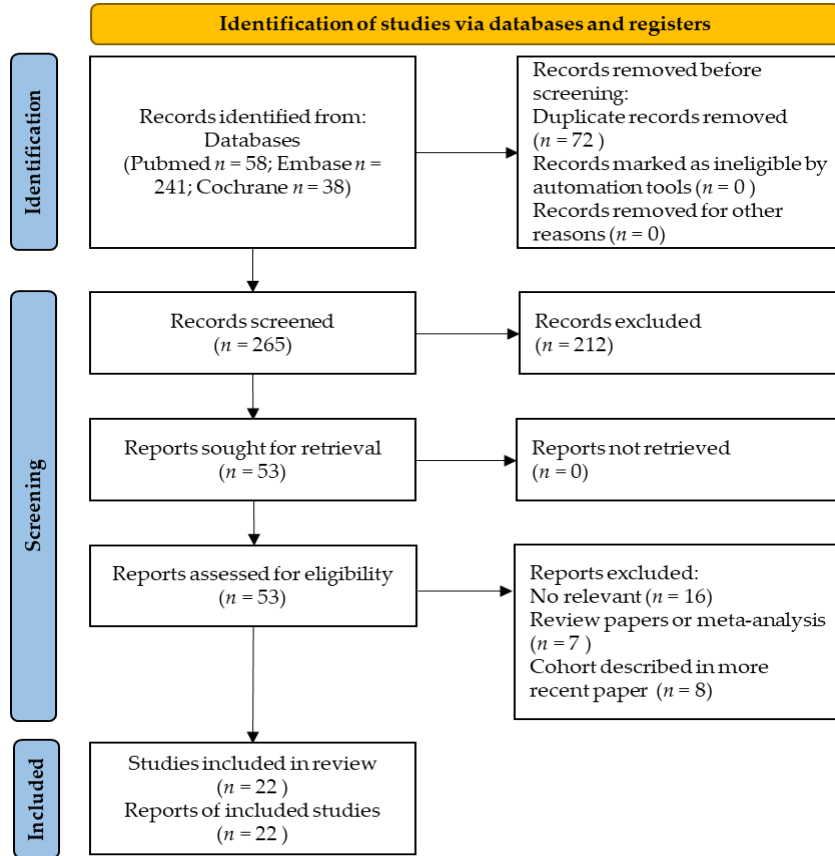
Preferred Reporting Items for Systematic Reviews and Meta-Analyses (PRISMA) flow diagram for study selection.

**Figure 2:**
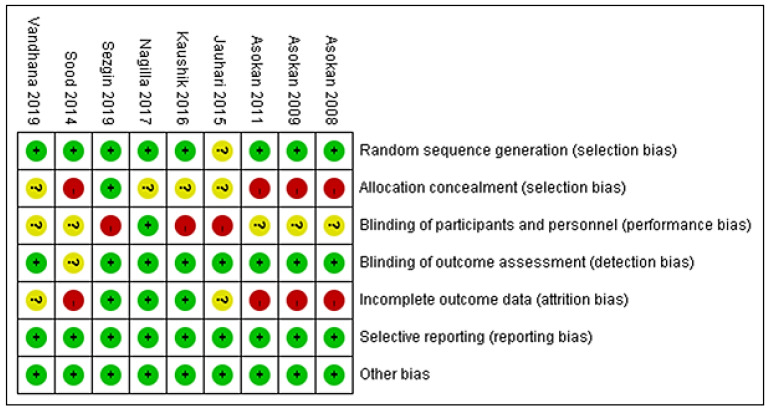
The risk-of-bias assessment results of the nine included trials.

We identified 253 records from the electronic databases. Twenty-seven studies were removed due to duplication. After the exclusion of duplication studies, a total of 226 records were screened, and 11 full-text articles were assessed for eligibility. One study was a single-arm study, and another study did not have related data and was excluded from this meta-analysis [3,15]. Finally, nine articles were selected for the qualitative review (Figure 1). The characteristics of these nine included studies are summarized in Table 1. There are five studies on oil pulling with sesame seed oil [8,16,17,18,19] and four studies with coconut oil [6,7,20,21]. Three studies used distilled or mineral water as a control group [7,20,21] and six studies compared the use of chlorhexidine with the coconut oil or sesame seed oil pulling intervention. All of the published data described patients treated between 2008 and 2019. The total number of subjects involved in these nine studies was 344. The risk-of-bias (ROB) assessment results of the nine included trials are summarized in Figure 2. The included studies varied in their risk of bias. Although the included articles are RCTs, there are still four articles that are low-quality studies after ROB assessment [8,16,17,19].

### 3.2. Statistical Analysis of Efficacy Outcomes

**Figure 3:**
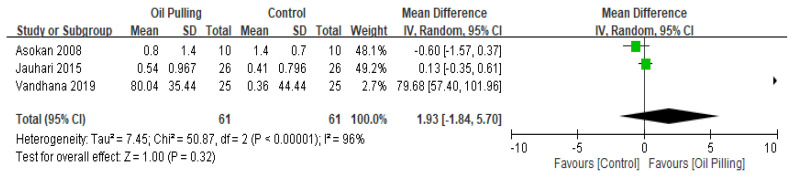
Forest plot for salivary *Streptococcus mutans* (SM) count.

**Figure 4:**
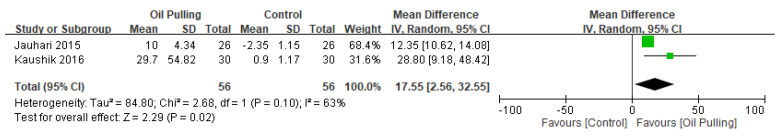
Forest plot for the salivary bacterial colony (BC) count.

Three studies included the salivary *Streptococcus mutans* (SM) count outcome and four studies reported data on the salivary bacterial colony (BC) count. Figure 3 shows a forest plot of the combined effects of oil pulling on salivary SM count. However, in salivary SM count, there was no significant difference between the two groups. The overall effect size for salivary SM count was 1.93, 95% CI −1.84, 5.7; *p* = 0.32. Figure 4 shows a forest plot of the combined effects of oil pulling on salivary BC count. There is a significant reduction in salivary BC count in the oil pulling group compared with the control group (MD: 17.55, 95% CI 2.56, 32.55; *p* = 0.02). The black diamond on the graph is the overall effect size, and the size of the green box is proportional to the study weight (the larger the square, the more accurate the study). The lines extending from either side of the center of the square are related to the CI.

**Figure 5:**
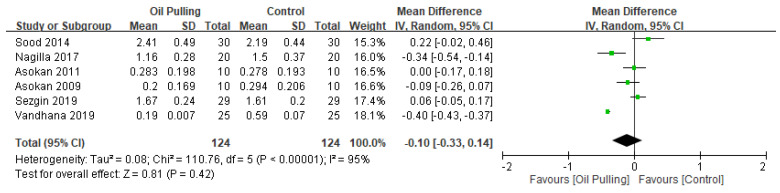
Forest plot for plaque index outcome.

**Figure 6:**
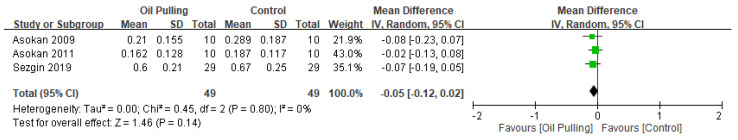
Forest plot for gingival index outcome.

In addition, six studies included post-intervention plaque index and four studies for post-intervention gingival index. Figure 5 showed the forest plot for the plaque index outcome. There was no significant difference between the oil pulling and control groups. The overall effect size for plaque outcomes was −0.10, 95% CI −0.33, 0.14; *p* = 0.42. However, we also found no significant difference in the gingival index score between the oil pulling group and the control group (Figure 6). The overall effect size for gingival index score was −0.05, 95% CI −0.12, 0.02; *p* = 0.41.

### 3.3. Publication Bias

**Figure 7:**
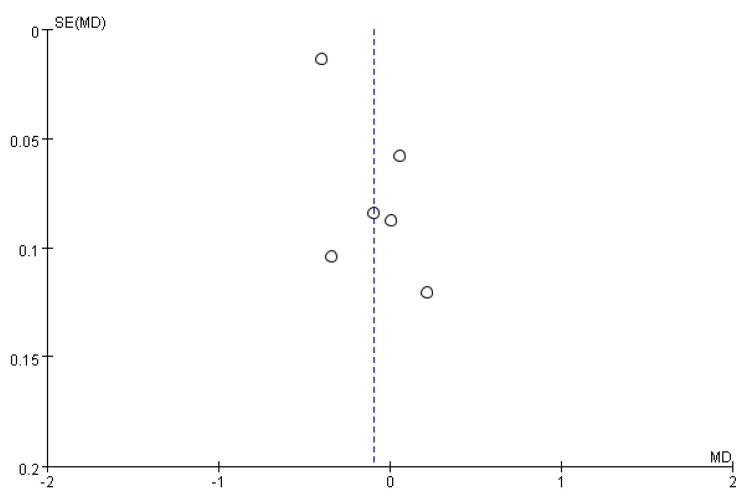
Funnel plot for plaque index outcome.

A visual inspection of the funnel plot of MD from these studies revealed asymmetry (Figure 7).

## 4. Discussion

The results of this meta-analysis indicate that oil pulling significantly reduces salivary BC count compared to water or chlorhexidine. However, in salivary SM count, there was no significant difference between the two groups. The results of this meta-analysis do not show any significant difference in plaque index and gingival index score. In a previous study, they aimed at a high-resolution examination of the oral microbiome dependent on oil pulling. They found that the pulling showed no significant preferences for particular bacteria, even considering morphology, cell wall structure, and oxygen tolerance. Therefore, they found a uniform reduction in overall microbial load [22]. Similar results were found in our study. Our study found that oil pulling did not significantly reduce salivary SM count, while oil pulling could significantly reduce salivary BC count.

Evidence shows that oil pulling can reduce total oral bacterial counts and reduce plaque and gingival scores. In addition, it reduces susceptibility to caries from marked to mild or moderate [16,23,24]. The exact mechanism of oil pulling is not clear. There are three possible mechanisms, one being the alkaline hydrolysis of the fat, leading to the process of saponification or “soap making”. Since the oil used in pulling contains fat, the alkaline hydrolysis process emulsifies the fat into bicarbonate ions, which are usually present in saliva. Soap is an effective cleaning agent that is mixed in the oil, thus increasing the surface area of the oil, which in turn increases the cleaning action [25]. Another theory is that the viscous properties of the oil inhibit plaque accumulation and adherent bacteria [8,26]. A third theory posits that the antioxidants present in the oil affect detoxification by preventing lipid peroxidation, and producing antibiotic-like substances, thereby contributing to the destruction of microorganisms and enhancing the effects of vitamin E in the oral cavity [25].

A systematic review of studies reviewed the effects of coconut oil for oil pulling on dental health and oral hygiene [9], and finally included 4 randomized trials [6,7,20,21], which included patients treated from 2015 to 2019. Of 182 patients, the intervention group used coconut oil, and the control group used distilled water or 0.2% chlorhexidine, and oral hygiene was different for each study. Two studies recommend oil pulling for 10 min [7,20], but one of them recommends not brushing [7]. One study recommended oil pulling for 15–20 min [20] while another study did not set a time limit but recommended oil pulling twice a day [21]. Most studies were 14 days in duration, with only one study being a 7-day study [7]. In a systematic review and meta-analysis in 2021, a total of nine studies were included for analysis, and the study results showed that oil pulling can effectively reduce bacterial colonization [10]. However, this systematic review indicates a significant statistical difference in plaque and gingival index between individuals with and without using coconut oil [10]. The results of this study are somewhat different from our results. This study is on the effect of using coconut oil for oil pulling on oral health. In our study, we included common conventional oils for oil pulling and conducted a literature search. For sunflower oil, olive oil, and corn oil no literature related to oil pulling was found in our literature search. Finally, we investigated the efficacy of coconut oil and sesame seed oil for oil pulling. We included nine RCTs in our study, and the results showed that oil pulling was only effective in reducing the salivary BC count. This is different from previous research.

This study has some limitations. First, this study did not obtain data from unpublished trials, which may have led to some publication bias. Second, the times and duration of oil pulling were not consistent across studies that were included in this analysis. Third, the number of included studies was small, and three of the studies were reported by the same author [8,16,17], which may introduce biases in outcome assessment. Our study found that common conventional oils (coconut oil and sesame seed oil) can reduce salivary BC count, and we further speculate that it may improve oral health. In addition, the time of day of oil pulling and the duration of use may affect the proliferation of bacteria. Therefore, future research should be conducted to study the effects on oral health of different times of day and the duration of use of oil pulling in the mouth.

## 5. Conclusions

The present meta-analysis found that oil pulling may have possible benefits in reducing salivary BC count. However, oil pulling has no significant effect on plaque index outcome and gingival index score. Therefore, more evidence from well-designed, large-scale, randomized trials is needed to confirm these results.

## Figures and Tables

**Table 1:** The characteristics included RCTs examining the effect of oil pulling.

Author(s)	Year	Design	No. of Patients	Intervention/Oral Hygiene Adjustment	Control	Outcomes Measured
Jauhari et al. [21]	2015	RCT	52	Coconut oil/Oil pulling twice daily	Distilled water. Mouthrinse twice daily	1. Oral microbial levels 2. Streptococcus mutans level in saliva
Kaushik et al. [20]	2016	RCT	60	Coconut oil/Oil pulling 10 mL for 10 min	Distilled water. Mouthrinse 5 mL for 1 min	1. Microorganism total colony-forming units
Nagilla et al. [7]	2017	RCT	40	Coconut oil/Oil pulling 10–15 mL for 10 min No toothbrushing	Mineral water. Mouthrinse. No toothbrushing	1. Plaque index
Sezgin et al. [6]	2019	RCT	58	Coconut oil/Oil pulling 10 mL twice daily for 15–20 min	Chlorhexidine 0.2%. Mouthrinse 10 mL twice daily for the 30 s	1. Plaque index 2. Gingival index 3. Bleeding on probing 4. Stain index
Asokan et al. [16]	2008	RCT	20	Sesame seed oil	Chlorhexidine mouthwash	1. Streptococcus mutans count in plaque 2. Streptococcus mutans count in saliva
Asokan et al. [8]	2009	RCT	20	Sesame seed oil	Chlorhexidine mouthwash	1. Plaque index score 2. Modified gingival index score 3. Aerobic microorganism total colony count
Asokan et al. [17]	2011	RCT	20	Sesame seed oil	Chlorhexidine mouthwash	1. Marginal gingival index 2. Plaque index
Vandhana et al. [18]	2019	RCT	50	Sesame seed oil	Chlorhexidine mouthwash	1. Salivary Streptococcus mutans count: 2. Plaque index
Sood et al. [19]	2014	RCT	60	Sesame seed oil	Chlorhexidine mouthwash/placebo	1. Plaque index 2. Gingival index

## Data Availability

The data presented in this study are available in the manuscript.
